# Vagueness in Medicine: On Disciplinary Indistinctness, Fuzzy Phenomena, Vague Concepts, Uncertain Knowledge, and Fact-Value-Interaction

**DOI:** 10.1007/s10516-021-09573-4

**Published:** 2021-07-05

**Authors:** Bjørn Hofmann

**Affiliations:** 1 Institute for the Health Sciences at the Norwegian University of Science and Technology (NTNU) at Gjøvik, PO Box 1, 2802 Gjøvik, Norway; 2 Centre of Medical Ethics at the University of Oslo, Oslo, Norway

**Keywords:** Vagueness, Uncertainty, Borderline, Medicine, Fact-value

## Abstract

This article investigates five kinds of vagueness in medicine: disciplinary, ontological, conceptual, epistemic, and vagueness with respect to descriptive-prescriptive connections. First, medicine is a discipline with unclear borders, as it builds on a wide range of other disciplines and subjects. Second, medicine deals with many indistinct phenomena resulting in borderline cases. Third, medicine uses a variety of vague concepts, making it unclear which situations, conditions, and processes that fall under them. Fourth, medicine is based on and produces uncertain knowledge and evidence. Fifth, vagueness emerges in medicine as a result of a wide range of fact-value-interactions. The various kinds of vagueness in medicine can explain many of the basic challenges of modern medicine, such as overdiagnosis, underdiagnosis, and medicalization. Even more, it illustrates how complex and challenging the field of medicine is, but also how important contributions from the philosophy can be for the practice of medicine. By clarifying and, where possible, reducing or limiting vagueness, philosophy can help improving care. Reducing the various types of vagueness can improve clinical decision-making, informing individuals, and health policy making.

## Introduction: In Medicine We Trust

Health care in general, and medicine in particular, plays a key role in modern western societies. Large parts of most countries’ GNPs are used for health care and health professionals have a prominent standing in many countries.

Ever more phenomena have been made the subject matter of medicine (medicalization) and ever more conditions have been addressed by health care (risk factors, precursors, predictors, indicators) (Hofmann 2019a, b). Technological and scientific progress in a wide range of sciences have vastly increased the knowledge in medicine. Most recently, fields like machine learning, BigData, and synthetic biology have evoked a strong belief in precision medicine (PM) to increase the knowledge about and actionability with respect to health and disease in individuals as well as populations.

Despite great efforts and achievements, vagueness prevails in medicine: from the patient-physician encounter, to the diagnosis of disease, the treatment decisions and outcomes, and to the health of the patient. Despite high hopes and lofty hype of precision medicine and other approaches, massive results from such efforts are still called for (Pletcher & McCulloch 2017; Prasad 2016; Saracci 2018; Wilkinson et al. 2020).

In this article, I will provide an overview over vagueness in medicine. While there are many types of vagueness in the medical sciences, such as pathology and microbiology, and in various specialties, such as psychiatry (Keil et al. 2017), this article will focus on five overarching kinds of vagueness in medicine: disciplinary, ontological, conceptual, epistemic, and fact-value-related vagueness.

The objective of this is twofold. First, the review of the various kinds of vagueness in medicine illustrates how complex, challenging, and interesting the field of medicine is. Second, it also demonstrates how important contributions from philosophy can be for the practice of health care. By clarifying and, where possible, reduce or limit vagueness, philosophy can contribute to improved care. Awareness of the various types of vagueness is key to clinical decision-making, informing individuals and the public, and health policy making.

### Definition of Two Key Concepts

Vagueness is defined in terms of something to possess of borderline cases (Sorensen 2001) making it difficult to decide to what degree something (a thing or an instance) falls within a conceptual category (Hampton 2007). I do acknowledge that what we mean by vagueness is to some extent itself vague (Hu 2017). That is, ‘vagueness’ in ‘vagueness in medicine’ is vague. However, for the purpose of this study I will apply the standard definition of vagueness and focus on the various types of vagueness at play in the activity which falls under ordinary use of the concept of medicine.

As this manuscript is written during the SARS-COV-2 pandemic, the pandemic also is a tangible reminder of uncertainty and vagueness in medicine.

## Vagueness in Medicine

### Medicine as a Vague Discipline

Initially it is important to acknowledge that medicine is not a well-defined discipline. It builds on a great variety of other subjects, such as biology, chemistry, pathology, physics, and physiology. It is not always clear what belongs to medicine and what belongs to other disciplines. This leaves a lot of borderline cases. Adding new emerging disciplines, such as machine learning, BigData analysis, and synthetic biology to the plethora of disciplines increases borderline cases. Moreover, the unclear borders of the various fields comprising medicine adds to this vagueness.

Additionally, medicine is considered to be both a theory and a practice, and as a practice it is both clinical and experimental. As such there are number of borderline cases between (pure) theory and (pure) clinical practice. As there are no pure cases, it can be argued that medicine is a fallible endeavor (Gorovitz & MacIntyre 1976).

Furthermore, medicine has many goals (Hanson & Callahan 2000), and the borders between them are not clear. Medicine aims at curing, preempting, predicting, palliating, and understanding disease, as well as caring for the wellbeing of the person (Broadbent 2019).

Hence, medicine is in itself a vague discipline. Adding new, and supposedly precising disciplines, may not reduce or omit this fact. Nonetheless, philosophy can contribute to clarify the disciplines of medicine, and their borders, and demarcate the science of medicine (Varga 2021).

However, even if the discipline of medicine is vague, the things that it deals with do not have to be vague.

### Ont(olog)ical Vagueness: Continuity

Medicine deals with individual persons with unique life stories and exclusive biological, physiological, mental, and social makeup. Even well-described and well-defined diseases appear differently in individuals. Therefore, the subject matter of medicine, patients, is characterized by a having wide range of borderline cases, and as such fall under the definition of vagueness (Sorensen 2001).

Moreover, the basic phenomena of medicine, such as pain, suffering, harm, but also wellness and wellbeing are vague phenomena. For example, pain comes in many kinds and grades and is hard to assess. The same does suffering (Hofmann 2017) and wellbeing (Bester 2020). Moreover, according to Worall and Worall the nature of disease is unclear: “there is no such thing as disease. The notion fails to find any joints in nature at which to carve.” (Worall & Worall 2001, p.51). According to this way of thought, there is no disease in nature, only human significance attached to certain conditions (Sedgwick 1973). Hence, either one believes that diseases come in natural kinds or as social constructions, they appear on continuums, with numerous borderline cases, and as such are vague.

The challenge of continuity is visible with respect to other (and more technical) phenomena in medicine, such as dysfunction, symptoms, and signs. What counts as a sign of disease in one context, such as an enlarged heart, can be a sign of wellness and high performance in another, e.g., in an athlete. Even more, it is argued that “many medical objects and subjects, e.g., cells, tissues, organs, organisms, persons, patients, symptoms, diseases, individual disease states of patients, and recovery processes are ontically vague to the effect that their vagueness is principally not eliminable.” (Sadegh-Zadeh 2015).

Hence, many of the basic phenomena (that are classified, investigated, and manipulated) in medicine are vague. So are the phenomena that comprise medicine’s goal (health, welfare, wellbeing). Therefore, there is a basic vagueness inherent in the theory and practice of medicine. Again, philosophy of medicine and philosophy of science can contribute significantly in reducing the ontological vagueness, for example in delimiting basic phenomena, such as harm (McGivern & Sorial 2017), pain, pleasure, suffering (Schleifer 2014) and wellbeing.

### Conceptual Vagueness

In addition to and partly related to ontological vagueness, medicine is haunted by conceptual vagueness. Basic concepts, such as health and disease do not have clear boundaries, are based on other vague concepts (such as wellbeing and happiness), and are hard to define (Brülde 2000; Campbell et al. 1979; Doust et al. 2017; Ereshefsky 2009; Hofmann 2010; Merskey 1986; Parsons 1958; Schwartz 2017; Temple et al. 2001; Worrall & Worrall 2001). Thus, there are ample borderline cases (Hofmann 2005).

Accordingly, it has been maintained widely that disease is a vague concept (Nordby 2004; Rosenberg 1991; Sadegh-Zadeh 1980).”The meaning of the word’disease’ is vague, elusive, and unstable. You may reach for it, but you won’t grasp it, except in pieces and fragments. It is prone to changes, permutations, and shatterings—according to the circumstances, or irrespective of them.” (Sundström, 2001) The same claim of vagueness goes for other health related concepts, such as sickness and illness (Fulford 1989).

In general, a concept is considered to be vague if no additional criteria will improve its definition and sharpen its boundaries (Williamson 1994). Vague concepts have borderline cases that resist investigation (Sorensen 2001). In a *relative borderline case*, the concept (of disease) may be clear, but the means to decide whether or not a condition falls in under the concept (i.e. whether it is disease) are incomplete. In an *absolute borderline case*, no amount of conceptual analysis or empirical studies can settle the issue. Accordingly, conceptual vagueness can be due to confused perception of a real concept or because of vagueness in its extension: “first….that ‘disease’ is indeed a real concept, a ‘natural kind,’ but the perception of this real concept even amongst experts is vague and confused. …[or] second … that there is no real distinction in nature between ‘disease’ and ‘non-disease’.”(Worall & Worall 2001, p.49).

In medicine, a wide range of controversial cases have been discussed, such as baldness, skin wrinkles, cellulitis, freckles, jet lag, ear wax accumulation, teeth grinding, chronic fatigue syndrome, and fibromyalgia (Smith 2002). More recently, gender identity disorder and gender incongruence have been heatedly debated (Drescher 2014; F. Beek 2016; Poteat et al. 2019) and classified in the International Classification of Disease (ICD-11) amongst “conditions related to sexual health.”(F. Beek et al. 2016).

However, it is not only the basic concepts of medicine, such as health and disease that are vague, but many other concepts as well (Sadegh-Zadeh 2015). Deciding what falls under concepts like pneumonia, schizophrenia, and Alzheimer’s disease is not easy as there are plenty borderline cases. For example, persons may have an inflammation of the lung tissues, but no symptoms, or they may have symptoms but no inflammation. There is a continuum between these cases. Moreover, medical descriptions include vague predicates (pain, headache, icterus), quantifies (few, many, most), temporal notions (acute, chronic, rapid), and vague frequency notions (commonly, usually, often) (Sadegh-Zadeh 2015).

Take cancer as an example. It is defined by the National Cancer Institute as “[a] term for diseases in which abnormal cells divide without control and can invade nearby tissues” (https://www.cancer.gov/). The definition is vague, as it is unclear what “can invade nearby tissue” means. This has made precursors defined as cancers, such as ductal carcinoma in situ (DCIS). While the cells in DCIS may divide without control, they do not necessarily invade nearby tissue (Esserman et al. 2014), creating borderline cases. Practically, people can die with the condition and not from it (due to overdiagnosis). This has led to a reclassifying or renaming of diseases, for example from DCIS to Indolent Lesions of Epithelial Origin (IDLE) in order to avoid the “carcinoma” name (Esserman et al. 2009; Esserman & Varma 2019).

Because key concepts in medicine have practical implications, it is crucial to clarify their boundaries. For example, whether your condition falls under the concept of disease decides whether you get attention and care from health providers, whether you are freed from social obligations (to work), and whether you will get economic support (sickness benefits). Setting such limits have been called the line-drawing problem and is widely discussed in the philosophy of medicine (Hofmann 2021; Rogers & Walker 2017; Synnott 2002).

Hence, conceptual vagueness is a key challenge in medicine on many levels. Let us look closer at some sources of conceptual vagueness, and some suggested solutions: interrelated concepts, polyvalent concepts, and the expansion of concepts.

#### Different Concepts of Malady

One source of elusiveness in medical semantics is the imprecision and ambiguity of terms. Disease, illness, sickness, impairment, disorder, and dysfunction are frequently used interchangeably. However, some clarity has been provided in differentiating between illness, disease, and sickness. *Illness* has been defined as the personal experience of a (mental or bodily) condition that is considered to be harmful while *disease* are conditions that health professionals consider to be harmful or disadvantageous to a person (called patient), and *sickness* is the social role attributed to a person assumed to have a disease or being ill (Clouser et al. 1997; Hofmann, 2002a, 2011). All three fall under the concept of malady (Hofmann 2016b).

**Fig. 1:**
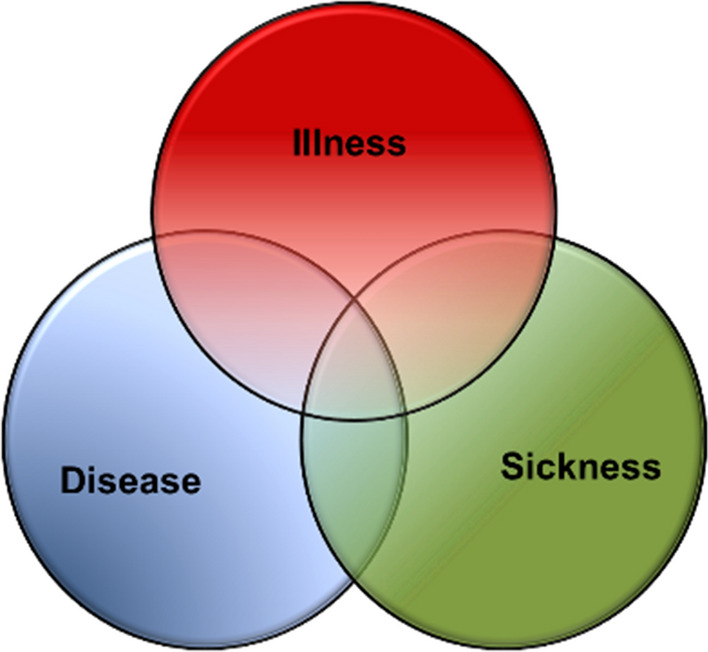
Three perspectives on human malady. Adapted from (Twaddle, 1994; Hofmann, 2002a)

The relationship between the three basic perspectives on human malady is illustrated in Fig. 1 illustrating how the terms are interrelated and partly interdependent (Hofmann 2002a). However, they only partly overlap, resulting in both in disagreement between the three perspectives on human malady and generating borderline cases.

The distinctions of the concepts of disease, illness, and sickness can explain many misunderstandings between health professionals and patients. For example, the physician can say that ‘I cannot find disease’ (meaning ‘I cannot identify any known diseases to the condition that you have’) while the patient hears “you are not ill” (meaning that ‘I do not believe that you experience illness’). Clarifying such misunderstanding can thus be of great value. 

#### Polyvalent Concepts in Medicine

While the distinction between disease, illness, and sickness is helpful in avoiding confusion, the terms are not always used consistently. For example, in psychiatry diseases are frequently called ‘disorders’ and are classified as ‘illnesses’ (Bolton 2008; Brülde 2010; Hacking 1998; Stein et al. 2010; Wakefield 1992). Hence, there is still ample bewilderment.

Another source of confusion is the fact that health related terms, such as disease, are used in many ways. When a person says that ‘I have a disease’ (when talking about his coughing), he uses the term ‘disease’ to explain why he is coughing. However, when the person maintains that ‘chronic fatigue syndrome is a disease,’ he uses it to claim that the condition described as CFS gives certain rights. Correspondingly, when a person says ‘I feel sick’ or ‘we live in a sick society’ the person means different things with ‘sick.’

Another reason why the concept of disease has been conceived of as ‘vague’ is that it can be understood in different ways, for example depending on knowledge and competence of the concept possessor (Burge 1991). The physician and the patient may have different understandings of ‘arthritis’ (Burge 1996). The physician knows much more about the pathophysiological conditions related to ‘arthritis’, and the patient more about the first-person experience of ‘arthritis.’ Accordingly, the vagueness is claimed to appear because people understand basic concepts, such as ‘disease,’ differently. In her study of atherosclerosis Mol (2002) found that the disease appears as many things in many contexts and refers to “a coexistence of multiple entities with the same name” (Mol 2002). Accordingly, there are many borderline issues between the different understandings of such basic concepts in medicine.

Another source of unclarity is the lack of conceptual unity. As argued by Kendell: “Most physicians … use the words disease and illness in different senses at different times.”(Kendell 1975) Correspondingly, Carl Jaspers has claimed that disease is not unitary concept, but that there exist many concepts of disease, which in principle can be sharp, but are not in practice (Jaspers 1913/1973). While some philosophers of medicine have been concerned with one conception of disease, for example the pathological concept of disease (Boorse 1993), they have willfully ignored others, such as clinical, judicial, and insurance-related notions (Worall and Worall 2001). Others have conceived of concepts such as health and disease “as a ‘family’ of concepts” (Stempsey 2006) or as models for searching for “unnoticed causal factors and expressions of disease” (Engelhardt and Wildes 1995). The point is that these conceptions are not unitary and leave lots of borderline cases.

Another level of confusion stems from the attempt to address different value settings (Stempsey 1999), perspectives (Hofmann 2002a), and theoretical aspirations (B Hofmann 2001a, b). Accordingly, it is argued that”at the present time, there is no unified concept of disease … In diagnosing different diseases, we often use entirely different types of fundamental criteria. We may have a concept of disease … based upon gross anatomical defects, microscopic anatomical changes, so-called specific etiological agents, specific deficiencies, genetic aberrations, physiological or biochemical abnormalities, constellations of clinical symptoms and signs, organ and system involvements, and even just descriptions of abnormalities.” (Engle and Davis 1963).

In sum, it can be argued with HG Wells that most terms are vague: “… every term goes cloudy at its edges… Every species waggles about in its definition, every tool is a little loose in its handle, every scale has its individual.” (Wells 1908).

While precisification can reduce or remove ambiguity and explanation can clarify complexity, conceptual vagueness may still leave many borderline cases (Sorensen 2001), which can confuse the communication between health professionals and patients (Nordby 2005).^1^ Attempts have been made to address such challenges, e.g., by defining disease as a concept of fuzzy disease (Sadegh-Zadeh 2000) based on the prototype resemblance theory of disease (Seising 2006). However, lots of vagueness still prevails.

#### Expanding the Borders of Medicine: Expansion of the Concept of Disease

As vagueness is defined in terms of borderline cases and one of the crucial and delimiting concepts in medicine is disease, it is especially interesting to look at the border of this concept. Moreover, it is widely argued that this border has been expanded significantly (Aronowitz 2009; Doust et al. 2017; Kaplan 2009; Moynihan et al. 2019). Table 1 summarizes six ways that the concept of disease has expanded and examples of how this extends its vagueness.

**Table 1:** Six types of expansion of the concept of disease with descriptions, examples, and kinds of vagueness. Adapted and expanded from (Hofmann 2019a, b)

Type of expansion	Description	Example	Kind of vagueness
Medicalization	Including ordinary life experiences (Conrad 2007)	Grief, sexual orientation (homosexuality)	Ontological vagueness
Overdiagnosis	Labelling indolent conditions as disease (Welch et al. 2011)	Ductal carcinoma in situ (DCIS)	Conceptual and epistemic vagueness
Aesthetic expansion	Treating aesthetic characteristics as disease (Hofmann 2019a, b)	Protruding ears	Conceptual vagueness
Pragmatic expansion	Making something disease because it can be detected and treated (Hofmann, 2002b)	Primary (essential) Hypertension, hyperglycemia	Epistemic vagueness
Conceptual expansion	Expanding definitions of disease (Caplan 2017)	Pre-diabetes, pre-Alzheimer, making menopause or aging a disease	Conceptual vagueness Epistemic vagueness
Ethical expansion	Making something disease because that will provide attention and access to care	Obesity (Hofmann 2016a), Attention Deficit Hyperactivity Disorder (ADHD), gender incongruence (Burke 2011)	Fact-value-vagueness

This article only leaves room for mentioning one type of expansion in some detail. The concept of disease was originally intrinsically defined by manifest conditions (such as visible infections and palpable tumors) and related, observed, and/or expressed suffering. Moreover, diseases were given names that were called ‘diagnoses.’

However, with time (and expanded knowledge) many conditions where defined as disease without manifest conditions or observed suffering. For example, precursors, risk factors, predictors, and indicators were included to fall under the term disease. Moreover, conditions were called disease beyond what was experienced as disadvantageous or harmful (Harris 2019; Harris et al. 2014).

Having a diagnosis has been extended beyond having a disease and having a disease has been expanded beyond what was observed or experienced. This has vastly extended the borderline cases of medicine, and hence, its vagueness.

While this short overview cannot go into details on all the conceptual vagueness, it suffices to illustrate the profoundness of the vagueness and that it has vast implications for individual persons, health professionals, health policy makers, groups of persons, and society at large. Philosophers have engaged not only in clarifying the basic concepts, such as health and disease, but also in in defining specific diseases and disease descriptions, and demarcating, delimiting, and define other concepts. Despite massive efforts, no general agreement is obtained, for example on clear definitions of health and disease. Accordingly, there is still much work to be done.

### Epistemic Vagueness: Uncertainty

Yet another major source of vagueness in medicine stems from uncertainty. Patients are uncertain about their health status, the description of their experiences (symptoms), of the information they get, about their health-related choices etc. Health professionals are uncertain about their tests (appropriateness, accuracy, actionability), about diagnoses, prognosis, as well as treatment options and outcomes (Hansson 1996). Moreover, they are uncertain about the basis of the knowledge, e.g., the premises and quality of the evidence (e.g., models, bias, study design). Correspondingly, health policy makers are uncertain about evidence, options, and outcomes of decisions, just to mention a few. As eloquently summarized by Voltaire: “Doctors are men who prescribe medicines of which they know little, to cure diseases of which they know less in human beings of whom they know nothing.” (Strauss 1968, p. 394).

Hence, uncertainty in medicine partly results from the types of vagueness discussed above, but also from ample borderline cases in the production and application of knowledge. There is an elaborate typology of epistemic uncertainty in medicine (Djulbegovic et al. 2011; Han et al. 2011; Hatch 2017), and this is not the place for a comprehensive review. The point here is only to highlight some basic types of uncertainty that pose fundamental challenges to medicine with substantial practical implications, and where the philosophy (of medicine and science) can contribute constructively.

Four basic types of uncertainty are: risk, fundamental uncertainty, ignorance, and indeterminacy (Van Asselt 2000; Wynne 1992). *Risk* is when you know certain outcomes and the chance that they occur. Given certain findings in a diagnostic test and the test characteristics (sensitivity, specificity), you know the risk that the patient has a given disease. Correspondingly, the chance of specific benefits and harms of various examinations and treatments is known, and hence the corresponding risk. The same goes for the chance of certain diseases to develop in specific manners (prognosis) and the chance of getting certain diseases for various groups in the population (epidemiology). However, even if you know the risk of a certain event, you do not know what will happen to the specific patient (the challenge applying class probability to individual cases).

According to *fundamental uncertainty* (also known as severe or Knightian uncertainty) you know about the outcome (e.g., the outcome of a specific disease) but you do not know the probability (distribution). For example, the chance of specific outcomes of diagnostics and treatments may be uncertain. While you have certain signs and symptoms, you do not always know whether they will develop to manifest disease and suffering (progression uncertainty). Moreover, you may also not know the chances that specific indicators, such as precursors, predictors, or risk factors, will develop into manifest disease (development uncertainty). This results in both underdiagnosis and overdiagnosis. Additionally, diagnostic tests frequently result in incidental findings of unknown implications.

*Ignorance* are unknown factors that are relevant for the diagnostic, prognostic, or therapeutic process, but which the health professional is not aware of. Before Wilhelm Conrad Röntgen’s discovery in 1895 people were ignorant of X-rays as medical doctors were of the side-effects of the drug thalidomide. Ignorance can be due to unknown effects of treatments (good or bad), unknown meaning of certain markers for diagnosis or prognosis, and due to the unknown relevance of individual health data.

*Indeterminacy*, which formally is a type of model validity uncertainty (or linguistic uncertainty), is uncertainty stemming from different ways to classify and categorize the medical phenomena. As discussed above, defining diseas(es), signs, and symptoms is not easy, and many (if not most) of the entities, phenomena, measures in medicine are vague. Defining and measuring personal experience (illness) is challenging. Moreover, there is vagueness in description of findings, in reporting diagnostic tests, and in interpreting and reporting treatment results. Correspondingly, it is difficult to define what is clinically relevant.

Table 2 gives an overview of these four types of uncertainty with examples.

**Table 2:** Four types of uncertainty classified according to outcomes and risks. Adapted from (Stirling 2010) and (Hofmann & Holm 2015)

Possibilities Probability	Known outcome	Unknown outcome
Known probability	**Risk** Test accuracy (sensitivity, specificity, predictive values) for the various examinations in different contexts Chance of specific benefits and harms of various examinations and treatments Chance of disease development (prognosis) Chance of getting certain diseases	**Indeterminacy** (Ambiguity, Vagueness) Defining diseas(es), signs, and symptoms How to define and classify specific entities, phenomena, measures Defining and measuring personal experience (illness) Vagueness in description of findings or in reporting of treatment results Defining clinical relevance
Unknown probability	**Fundamental** (Knightian) **Uncertainty** Unknown chance of specific outcomes of diagnostics and treatments Prognostic uncertainty Development uncertainty Progression uncertainty Overdiagnosis, underdiagnosis Incidental findings of unknown implications	**Ignorance** Unknown effects of treatments (good or bad) Unknown meaning of certain markers for diagnosis or prognosis Unknown relevance of individual health data

The various types of uncertainty have many sources and implications. They can be recognized as and resulting in vagueness in the production and application of medical knowledge and evidence. While risk, fundamental uncertainty, and ignorance can be reduced by scientific efforts, such as developing better methods (reducing risk), gaining knowledge about probability distributions (reducing fundamental uncertainty), and discovering new mechanisms (reducing ignorance), philosophy can make substantial contributions in identifying and clarifying the preconditions for the various types of uncertainty, and in particular, to address indeterminacy.

However, reducing epistemic uncertainty is clearly no easy task and fuels heated debates. While some argue that medicine is much less effective and that evidence is much weaker than we think (“medical nihilism”) demanding higher standards (Stegenga 2018), others confer to open conversations based on epistemic humility and valuing practice of principle in so-called medical cosmopolitanism (Broadbent 2019). Others again launch “deep medicine” using vast amounts of data to gain “deep” knowledge of individuals (Topol 2019). However, while reducing epistemic uncertainty is of outmost importance to patients, professionals, and policy makers, much more work is needed.

### Vagueness Due to Fact-Value Interactions

The last type of vagueness to be discussed in this article is a vagueness resulting from fact-value relationships. Many facts in medicine are value-related generating ample borderline issues. In diagnostics, disease definitions, and treatment outcome measures there are thresholds and cut-off values that are not given by nature but decided upon by professionals (and others) from what is believed to give most benefit (or profit).

For example, the various types of uncertainty discussed above result in tradeoffs between Type I errors (rejecting the null hypothesis when it's actually true) and Type II errors (failing to reject the null hypothesis when it's actually false). In particular, the cut-off values in diagnostic tests is a tradeoff between test sensitivity and specificity and depend on how you value false positive test results compared to a false negative ones.

Correspondingly, between normal and pathological there are great many borderline cases. Drawing the line for what is normal is not only a factual issue but involves conceptions of good and bad. While this is most obvious in psychiatry (Keil et al. 2017), the same goes for somatic medicine. There is no natural cutoff for hypertension in nature. Correspondingly, and as already alluded to, there are also a wide range of borderline cases between health and disease (Hucklenbroich 2017). People consider themselves healthy although they have many diagnoses and are on medications for several diseases.

Also, the production of evidence consists of value issues. For example, in deciding how much evidence is enough to accept or reject a hypothesis (Hempel 1965), choosing methodology (e.g., the setting of a level of statistical significance or choosing model organism) (Wilholt 2009), characterizing and assessing evidence, and interpreting data (Douglas 2017). This value-relatedness in the medical evidence-production can be seen in the field of health technology assessment where a wide range of (moral) value-judgments have been identified in every step of the production and assessment of evidence (Hofmann et al. 2018, 2014).

The vagueness due to the fact-value interrelatedness partly stems from many medical concepts being what Bernard Williams called ‘thick concepts’ (Williams 1985). According to Williams, thick concepts express a union of fact and value and have extensions which are co-determined by both their descriptive and their evaluative meaning. “Any such concept,…, can be analyzed into a descriptive and a prescriptive element: it is guided round the world by its descriptive content, but has a prescriptive flag attached to it. It is the first feature that allows it to be world-guided, while the second makes it action-guiding.” (Williams 2011, p.156) Following Williams you cannot decide on the extension of ‘disease’ based only on its descriptive content. The combination of descriptive and normative content makes some characterize it as vague (Stoecker & Keil 2016).

The vagueness stemming from the fact-value interaction, but also from conceptual and epistemic vagueness, can be recognized in the vagueness in diagnoses. Classifications of diseases (taxonomies, nosologies) are vague in the sense that they leave many borderline cases. A patient may not fully qualify for a specific diagnosis or be on the borderline of two or more diagnoses.

On the one hand the diagnostic criteria may be vague making it unclear whether a person falls under the diagnosis or not. On the other hand, the criteria may be clear, but it is unclear whether it applies to a specific person (e.g., due to uncertainty).

While paradoxes (Hofmann 2001a, b) and fuzzy reasoning has been suggested to address the vagueness in medical diagnosis (Seising 2006) others argue we should accept and even praise vagueness (Kwiatkowska 2013; Van Deemter 2012). As no agreement has been reached in the fact-value interactions, which are crucial for demarcating and defining care, more work to reduce this type of vagueness is urgently needed.

## Discussion

This article gives an overview over various types of vagueness in medicine and uses the case of precision medicine, which is branded a way to make medicine more precise, to illustrate that vagueness may well prevail. The point has been to show that vagueness in medicine warrants attention.

There are a number of limitations and shortcomings of this study. First, it does not take a stance on many of the philosophical issues in the literature on vagueness (Black 1937; Hu 2017; Kenney & Smith 1996; Russell 1923; Sorensen 2001). I have taken the standard definition of vagueness for granted. There is certainly much more to say about that.

Second, I have accepted that epistemic vagueness exists in terms of epistemic uncertainty. While some would count epistemic uncertainty as vagueness (Merricks 2001), others are more hesitant (Sorensen 2001). In this section I have taken as a point of departure that the production of medical knowledge and evidence involves and leaves a wide range of borderline cases. The debate on the status of epistemic vagueness is beyond the scope of this study.

Third, the study of medical vagueness is by no means exhaustive or exclusive. There are certainly many types of vagueness in medicine that are not addressed in this article. As stated at the outset, specific fields or specialties of medicine may have their own vagueness issues (Keil et al. 2017). Moreover, there is much more to say about each type of vagueness than has been presented here. The point here, however, has been to provide an overview in order to facilitate more specific in-depth studies.

Fourth, I have used a wide range of different examples. While most examples are from “traditional medicine” I would argue that the same kind of vagueness exists for new “paradigms” in medicine, e.g., “precision medicine” (Duffy 2015; Koenig et al. 2017; Vogt et al. 2016a, 2016b). However, a more detailed elaboration of this would merit a separate study.

Fifth, while I have emphasized the importance of reducing vagueness in medicine for patients, professionals, and policy makers, and I have highlighted the significance of philosophy for doing so, I have not gone in great detail in showing how philosophy can do so. The reason is that there are so many important issues that have no solutions yet, and that are still controversial. Discussing them all is beyond the scope of this article. Here the point has been to review various forms of vagueness, to indicate where solutions are urgently needed, and thereby hopefully to draw attention to and inspire efforts to solve them.

## Conclusion

In this article I have discussed five types of vagueness in medicine: disciplinary, ontological, conceptual, epistemic, and fact-value-related vagueness. First, medicine is a discipline with unclear borders, as it builds on a wide range of other disciplines and subjects. Second, medicine deals with a wide range of indistinct phenomena resulting in borderline cases. Third, medicine uses many vague concepts, making it unclear which situations, conditions, and processes that fall under them. Fourth, medicine is based on and produces uncertain knowledge and evidence. Fifth, vagueness emerges in medicine as a result of a wide range of fact-value-interactions.

The review of the various kinds of vagueness in medicine can help us understand and address many of the basic challenges of modern medicine, such as overdiagnosis, underdiagnosis, and medicalization. Even more, it illustrates how complex and challenging the field of medicine is, but also how important contributions from the philosophy can be for the practice of health care. By clarifying and, where possible, reducing or limiting vagueness, philosophy can help improving care. Reducing the various types of vagueness can improve clinical decision-making, informing individuals, and health policy making.

## Abbreviations

PM: Precision Medicine
GNP: Gross National Product

## Footnotes

1 I have not differentiated between linguistic vagueness (such as vague terms) and semantic vagueness (vagueness in representation). See for example reference Sadegh-Zadeh, K. (2015). *Handbook of Analytic Philosophy of Medicine* (Vol. 119). Springer.
